# Delayed encephalopathy after COVID-19: A case series of six patients

**DOI:** 10.1097/MD.0000000000031029

**Published:** 2022-10-21

**Authors:** Takayoshi Akimoto, Makoto Hara, Kenta Tasaki, Yusuke Kurosawa, Tadaharu Nakamoto, Satoshi Hirose, Tomotaka Mizoguchi, Yuki Yokota, Satoko Ninomiya, Hideto Nakajima

**Affiliations:** a Division of Neurology, Department of Medicine, Nihon University School of Medicine, Itabashi-ku, Tokyo, Japan; b Division of Respiratory Disease, Nihon University School of Medicine, Itabashi-ku, Tokyo, Japan.

**Keywords:** blood-, brain barrier, COVID19, encephalopathy

## Abstract

Acute encephalopathy is a severe neurological complication of coronavirus disease 2019 (COVID-19). Most cases of acute encephalopathy associated with COVID-19 occur within several weeks of COVID-19 onset. We describe a case series of 6 patients who developed delayed encephalopathy (DE) after COVID-19.

**Patient concerns and diagnoses:**

We evaluated patients who recovered from COVID-19 and showed acute disturbance of consciousness or focal neurological deficits without recurrence of pneumonitis. Six patients, 2 females and 4 males, with ages ranging from 65 to 83 years were included. Durations of hospitalization due to COVID-19 were between 25 and 44 days. The severity of COVID-19 was moderate in 5 and severe in 1 patient. Patients were rehospitalized for acute disturbance of consciousness concomitant with postural tremor and, abnormal behavior, hemiplegia, aphasia, or apraxia between 34 and 67 days after the onset of COVID-19. Chest computed tomography showed no exacerbation of pneumonitis. Brain magnetic resonance imaging showed no specific findings except in 1 patient with an acute lacunar infarction. Electroencephalogram demonstrated diffuse slowing in all patients. Repeat electroencephalogram after recovery from encephalopathy demonstrated normal in all patients. One of the 6 patients had cerebrospinal fluid (CSF) pleocytosis. CSF protein levels were elevated in all patients, ranging from 51 to 115 mg/dL. CSF interleukin-6 levels ranged from 2.9 to 10.9 pg/mL. The immunoglobulin index was 0.39 to 0.44. Qlim(alb) < QAlb indicating dysfunction of the blood–brain barrier was observed in all patients. Severe acute respiratory syndrome coronavirus 2 reverse transcription polymerase chain reaction of CSF was negative in all patients. Neuronal autoantibodies were absent in serum and CSF.

**Interventions and outcomes:**

Immunotherapy including steroid pulses was administered to 3 patients; however, symptoms of encephalopathy resolved within several days in all patients, regardless of treatment with immunotherapy, and their consciousness levels were recovered fully. Notably, postural tremor remained for 2 weeks to 7 months.

**Lessons:**

In our patients, DE after COVID-19 was characterized by symptoms of acute encephalopathy accompanied with tremor in the absence of worsening pneumonitis after the fourth week of COVID-19 onset. Our findings indicate blood–brain barrier dysfunction may contribute to the pathogenesis of DE after COVID-19.

## 1. Introduction

Coronavirus disease 2019 (COVID-19) is a disease caused by infection with the severe acute respiratory syndrome coronavirus 2 (SARS-Cov-2) and presents with a variety of organ disorders, predominantly involving the respiratory system. COVID-19 is also associated with a range of neurological complications.^[1–9]^ Acute encephalopathy is one of the neurological complications of COVID-19, which typically occurs in patients with greater severity of COVID-19, older age, acute respiratory distress syndrome, and multiple organ failure.^[1]^ Thus, most cases (90–92%) of acute encephalopathy develop within 3 weeks of COVID-19 onset.^[2,3]^ On the other hand, there have been reports of patients developing symptoms of acute encephalopathy, including impaired consciousness, memory dysfunction, and impaired verbal fluency, more than 4 weeks after the onset of COVID-19 ^[10,11]^; however, it was not clarified whether the pneumonitis had fully improved at the time of occurring encephalopathy in these reports.^[10,11]^ Furthermore, a disease known as long COVID-19 has been reported, which manifests as systemic symptoms weeks to months after COVID-19.^[12–14]^ Herein, we report 6 cases of encephalopathy distinct from long COVID-19 that developed more than 4 weeks after COVID-19 infection without exacerbations of pneumonitis.

## 2. Methods

We evaluated patients who recovered from COVID-19 pneumonitis and showed acute encephalopathy or stroke-like symptoms without recurrence of COVID-19 pneumonitis. The patients’ COVID-19 clinical courses, symptoms of neurological deficits, laboratory findings, magnetic resonance imaging (MRI) findings, and electroencephalogram (EEG) findings were reviewed. Serum C-reactive protein (CRP) level, D-dimer, blood cell count, sodium, calcium, blood glucose, ammonia, TSH, FT4, anti-nuclear antibody, anti-TPO antibody, anti-thyroglobulin antibody, anti-GAD65 antibody, vitamin B1, vitamin B12, rapid plasma reagin test for syphilis, cerebrospinal fluid (CSF) cell count, protein, interleukin 6 (IL-6), IgG index, oligoclonal band, and SARs-COV-2 reverse transcription polymerase chain reaction (PCR) were reviewed. Qlim(alb) and QAlb were calculated from CSF and serum. Qlim(Alb) was calculated as 4 + (age/15), and QAlb was calculated as CSF albumin[mg/dL]/serum albumin[g/L]. Qlim(alb) < QAlb indicates dysfunction of the blood-brain barrier (BBB).^[15,16]^ To investigate for an autoimmune etiology of central nervous system (CNS), in-house screening of neuronal antibodies in all serum and CSF was implemented with the following techniques: tissue-based assay (TBA) involving immunohistochemistry with rat brain frozen sections; and live-neuron assay involving immunocytochemistry with rat primary hippocampal neurons.^[17,18]^ Confirmation tests for intracellular (Hu, Yo, CV2, Ri, Ma2/Ta, GAD65, amphiphysin, recoverin, SOX1, titin, zic4, Tr) and neuronal surface antigens (NMDAR, LGI-1, AMPAR, Casper 2, GABABR, DPPX, IgLON5) relevant to CNS disorders were performed with line blots (EUROLINE, Euroimmun Germany) and cell-based assays (BIOCHIP, Euroimmun, performed by Labor Berlin). Informed consent was obtained from all patients or their families.

## 3. Results

**Figure 1.**
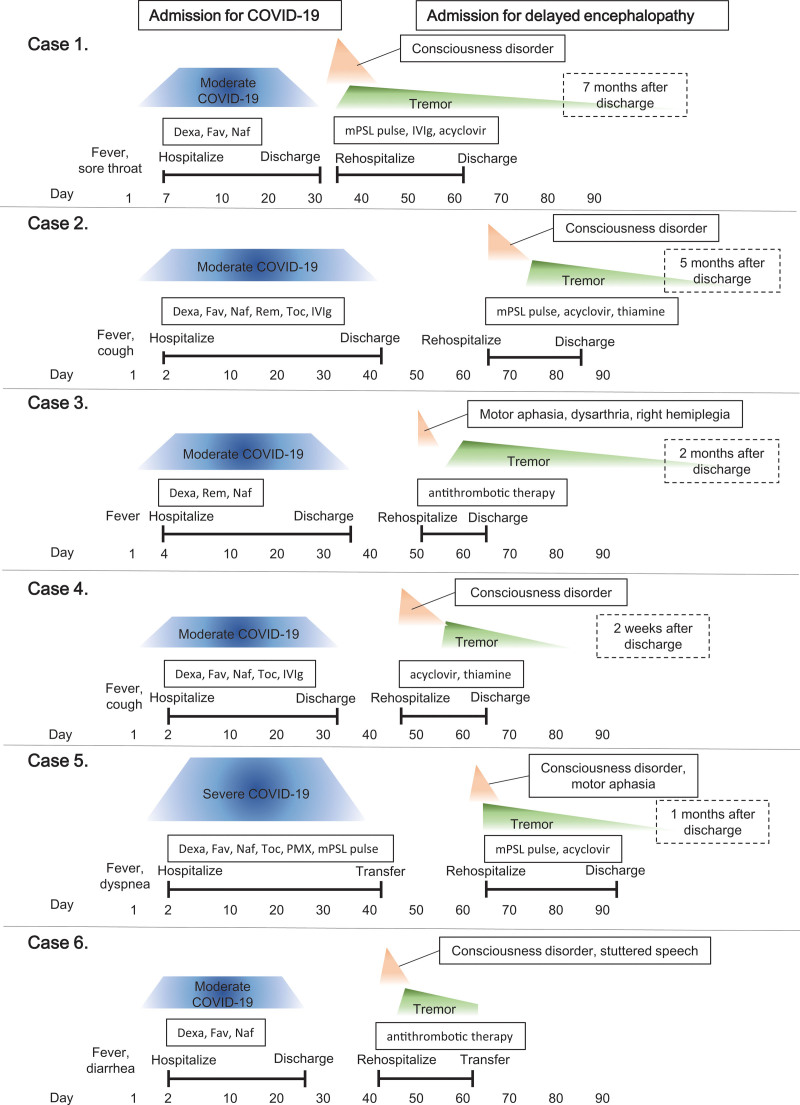
Clinical course of 6 patients with delayed encephalopathy after COVID-19. COVID-19 = coronavirus disease 2019, Dexa = dexamethasone, Fav = favipiravir, HTN = hypertension, IVIg = immunoglobulin, mPSL = methylprednisolone, Naf = nafamostat, PMX = endotoxin adsorption therapy, Rem = remdesivir, Toc = tocilizumab.

Six patients were considered as delayed encephalopathy (DE) after COVID-19. Clinical courses, laboratory data, MRI, and EEG findings are shown in Table 1. A schema of the clinical course of the 6 patients is shown in Figure 1. The detailed patients’ clinical course is described in Supplemental file 1, http://links.lww.com/MD/H580.

**Table 1:** Clinical courses, laboratory data, MRI, and EEG findings of 6 patients with delayed encephalopathy after COVID-19.

Case no.	1	2	3	4	5	6
Age (y)	65	68	72	83	73	77
Sex	Male	Female	Male	Male	Female	Male
Previous medical history	HTN, T2DM	Lung cancer (adeno)	Rectal cancer (adeno)	HTN, T2DM, Colon cancer (adeno)	None	Lung cancer (small cell)
Severity of COVID-19	Moderate	Moderate	Moderate	Moderate	Severe	Moderate
Treatment for COVID-19	Dexa, Fav, Naf	Dexa, Fav, Naf, Rem, Toc, IVIg	Dexa, Rem, Naf	Dexa, Fav, Naf, Toc, IVIg	Dexa, Fav, Naf, Toc, PMX, mPSL pulse	Dexa, Fav, Naf
Duration of admissionfor COVID-19(d)	25	44	36	31	44	25
Duration from COVID-19 toencephalopathy(d)	34	67	51	47	62	43
Symptoms other than tremor	Abnormal behavior, attention disorder, myoclonus	Inappropriate response, apraxia	Motor aphasia, right hemiparesis	Abnormal behavior, motor aphasia	Disorientation, motor aphasia	Stuttered speech, gait disturbance
Temperature (°C)	37.3	37.2	36.6	36.8	36.9	37.5
GCS on admission	12 (E4V3M5)	13 (E4V3M6)	14 (E4V4M6)	13 (E4V3M6)	13 (E4V3M6)	14 (E4V4M6)
Nasopharyngeal PCR	+	+	-	-	-	+
Serum CRP (mg/dL)	<0.1	0.49	<0.1	1.51	<0.1	0.43
Serum D-dimer (μg/mL)	<1.0	<1.0	8.7	1.8	<1.0	1.9
CSF WCC (mm^3^)	18	1	2	1	1	2
CSF protein (mg/dL)	115	63	97	61	51	61
CSF IL-6 (pg/mL)	10.9	7.9	3.2	2.9	N.A.	4.5
IgG index	0.45	0.44	0.44	0.41	0.42	0.39
Qlim (alb)	8.3	8.5	8.8	9.5	8.9	9.1
Qalb	22.3	10.8	20.1	11.9	9.9	12.7
Oligoclonal band	-	-	-	-	-	-
CSF SARs-COV-2 PCR	-	-	-	-	-	-
MRI	No abnormality	No abnormality	No abnormality	No abnormality	No abnormality	Acute lacunar infarction in right basal ganglia
1st EEG (d)^*^/findings	10/diffuse slowing	15/diffuse slowing	9/diffuse slowing with FIRDA	10/diffuse slowing with FIRDA	3/diffuse slowing with FIRDA	7/diffuse slowing with FIRDA
2nd EEG (d)^*^/findings	17/normal background rhythm	32/normal background rhythm	215/normal background rhythm	72/normal background rhythm	147/diffuse alpha background rhythm	
Treatment for delayed encephalopathy	mPSL pulse, IVIg, acyclovir	mPSL pulse, acyclovir, thiamin	Antithrombotic therapy	Acyclovir, thiamin	mPSL pulse, acyclovir	Antithrombotic therapy
Days from admission to onset of tremor	1	9	11	10	1	7
Approximate duration of tremor	7 mo	5 mo	2 mo	2 wks	1 mo	N.A.
Duration of admission for delayed encephalopathy (d)	24	20	37	19	30	20
mRS score at discharge	1	1	1	1	1	4

COVID-19 = coronavirus disease 2019, CRP = C-reactive protein, CSF = cerebrospinal fluid, Dexa = dexamethasone, EEG = electroencephalogram, Fav = favipiravir, FIRDA = frontal intermittent rhythmic delta activity, GCS = Glasgow coma scale, HTN = hypertension, IL-6 = interleukin-6, IVIg = immunoglobulin, mPSL = methylprednisolone, MRI = magnetic resonance imaging, mRS = modified rankin scale, N.A. = not available, Naf = nafamostat, PCR = polymerase chain reaction, PMX = endotoxin adsorption therapy, Rem = remdesivir, SARS-CoV-2 = severe acute respiratory syndrome coronavirus 2, T2DM = type 2 diabetes mellitus, Toc = tocilizumab, WCC = white cell count.

**Figure 2.**
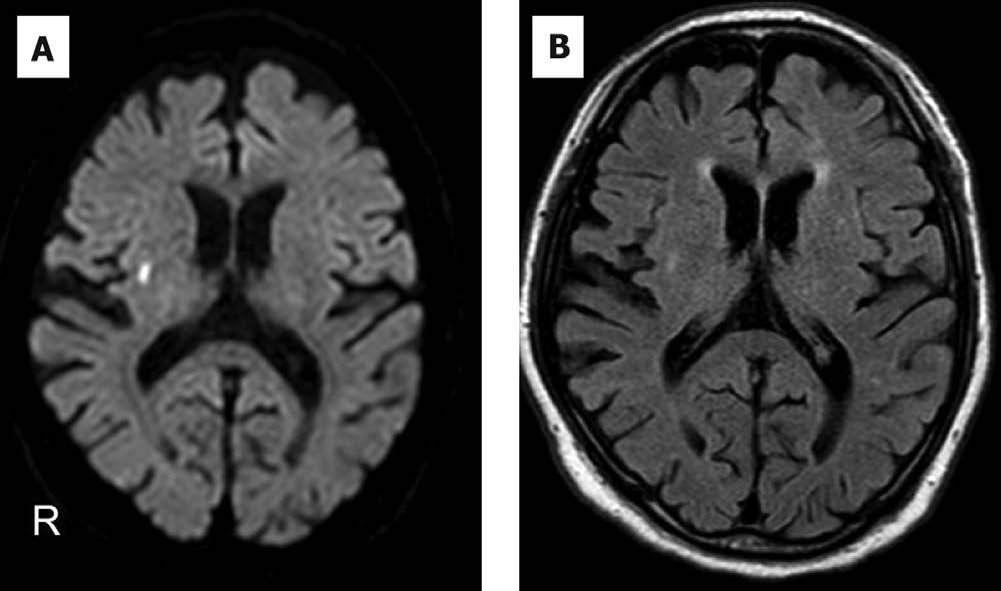
Brain MRI in Case 6 admitted with delayed encephalopathy (A, diffusion-weighted image; B, fluid-attenuated inversion recovery image). Acute lacunar infarction is seen in the right basal ganglia. MRI = magnetic resonance imaging.

The 6 patients, 2 of whom were female, ranged in age from 65 to 83 years. They were admitted to our hospital due to COVID-19 for 25 to 44 days and subsequently discharged home. The severity of COVID-19^[19]^ was moderate in 5 and severe in 1 patient. They were rehospitalized for acute neurological symptoms 34 to 67 days after the onset of COVID-19. The neurological symptoms were abnormal behavior in 2 patients (case 1, 4), motor aphasia in 3 patients (case 3, 4, 5), inappropriate response for questions and apraxia in 1 patient (case 2), hemiplegia in 1 patient (case 3), and stuttered speech and gait disturbance in 1 patient (case 6). Their temperatures ranged from 36.6 to 37.3 °C, and their Glasgow coma scale score ranged from 12 to 14 points on admission. Three of the 6 patients had positive nasopharyngeal SARS-Cov-2 PCR on admission for encephalopathy. Serum CRP was <0.1 to 1.51 mg/dL, and D-dimer was <1.0 to 8.7 μg/mL. One patient was positive for serum anti-thyroglobulin antibody. CSF examination showed pleocytosis in 1 patient (Case 1; 18/mm^3^, all mononuclear cells). CSF protein was elevated in all patients, ranging from 51 to 115 mg/dL. CSF IL-6 ranged from 2.9 to 10.9 pg/mL. The IgG index was 0.39 to 0.44, and oligoclonal bands were negative in all tested patients. SARS-Cov-2 PCR of CSF was negative in all patients. CSF cytology was performed in 2 cases (cases 4 and 6) with malignant tumors (cases 2, 3, 4, and 6), 1 in class I and class II. Screening for neuronal antibodies was negative in all patients. Qlim(alb) and QAlb were measured, and Qlim(alb) < QAlb was seen in all patients. Chest computed tomography (CT) demonstrated no exacerbation of COVID-19 pneumonitis in all patients. Brain MRI demonstrated no abnormal findings except in 1 patient with lacunar infarction in the right basal ganglia (Fig. 2.). A contrast-enhanced brain MRI was performed 1 month prior to admission for initial COVID-19 pneumonitis in 1 patient with lung cancer (case 6), but no tumor or meningeal contrast effect was observed. EEG performed 3 to 15 days after the onset of DE demonstrated diffuse slowing in all patients, with repeat EEG examinations demonstrating improvement after resolution of encephalopathy. Immunotherapy including steroid pulse therapy was administered to 3 of 6 patients. In all patients, acute cognitive disorders and focal neurological deficits recovered within several days to weeks, regardless of whether immunotherapy was administered. However, tremor developed in all patients and was persistent for 2 weeks to 7 months. During the observation period, 4 cases with malignant tumors (cases 2, 3, 4 and 6) did not exhibit symptoms suggestive of carcinomatous meningitis.

## 4. Discussion

Herein, we present a case series of 6 patients with DE after COVID-19. They showed acute encephalopathy more than 4 weeks after COVID-19 infection without exacerbations of pneumonitis. Qlim(alb) < QAlb, an indicator of BBB dysfunction, was observed in all patients. In addition to encephalopathy symptoms, tremor developed in all patients and was persistent for 2 weeks to 7 months.

Acute encephalitis or encephalopathy account for 21.3% to 51.0% of neurological complications of COVID-19.^[1,4–6,20]^ Severe COVID-19 is a known risk factor for encephalopathy, with most cases of acute encephalitis or encephalopathy associated with COVID-19 occurring in the acute phase of COVID-19 onset.^[1,2,5]^ According to multicenter study from Spain and Italy, encephalitis, encephalopathy, or acute disseminated encephalomyelitis occurring after four weeks from the onset of COVID-19 was 4 to 8%.^[2,3]^ Ishiyama et al reported that 10 of 12 patients who required ventilation due to COVID-19 developed encephalopathy 38 to 54 days (median, 51 days) after the onset of COVID-19.^[10]^ In those patients, brain dysfunction, memory disturbance, impaired verbal fluency, and dyscalculia were commonly observed, with tremor seen in 58% of patients.^[10]^ Reming et al reported a case of transient encephalopathy approximately 1 month after the onset of COVID-19, in which investigations demonstrated a CSF cell count of 3/mm^3^, total protein level of 76 mg/dL, and diffuse slowing on EEG.^[11]^ These studies indicate a proportion of patients are at risk of developing encephalopathy up to 4 weeks after the onset of COVID-19; however, it is not clarified in these reports whether COVID-19 pneumonitis had fully improved at the time when neurologic deficits were observed.^[2,3,10,11]^ In the present series, acute encephalopathy symptoms developed after recovery from COVID-19 without exacerbation of pneumonitis. In addition, the severity of COVID-19 was moderate or severe although no patients required mechanical ventilation, which differs from the previous report.^[10]^ The pathogenesis of acute neurological complications associated with COVID-19 has been posited to involve direct viral invasion of the CNS, para-infectious, post-infectious, or vascular disease.^[9]^ DE after pneumonitis has rarely been discussed, and to our knowledge, there have been only a few case reports of CNS complications following Legionnaires’ disease that occurred more than four weeks after the onset of pneumonitis.^[21,22]^ The pathogenesis of these cases was unknown, but it has been suggested that autoimmune or autoinflammatory processes induced by *Legionella* were involved.^[21,22]^ In our series, all but 1 patient (case 1) had normal CSF cell counts, although elevated CSF total protein levels were observed in all patients. Furthermore, we did not observe positive SARS-CoV-2 reverse transcription PCR testing, oligoclonal bands, higher IgG indices, increased CSF IL-6 levels, nor detection of neuronal antibodies related to autoimmune encephalitis in any patient, indicating autoimmune and autoinflammatory processes, including viral CNS infection, were unlikely to be involved in the pathogenesis of the present cases. At the time of hospitalization for DE, nasopharyngeal PCR for SARS-Cov-2 testing was positive in 3 patients. PCR testing can remain positive for several months after SARS-Cov-2 infection.^[23]^ Although no patients were observed to have worsening respiratory symptoms or chest CT findings, persistent ongoing infection could not be fully ruled out. Regarding vasculopathy, serum D-dimer levels were mildly elevated in 3 patients, and normal in 3 patients. Five of 6 patients had no evidence of acute ischemic or hemorrhagic stroke on brain MRI several days after the onset of neurological symptoms. One patient (case 6) was found to have an acute right lacunar infarction in the right basal ganglia associated with noticeable stuttering of speech. Stuttering can be a sign of ischemic stroke;^[24]^ however, it was unreasonable that bilateral tremor and diffuse slowing on EEG were derived from the lacunar infarction.

Qlim(alb) < QAlb, suggesting BBB failure, was identified in all our patients. SARS-Cov-2 is known to infect vascular endothelial cells via the angiotensin converting enzyme receptor, and it has been speculated that SARS-Cov-2 may be able to infect astrocytes, the main components of the BBB, and cause inflammatory injury to the BBB as astrocytes also express the ACE receptor.^[25]^ Neumann et al reported elevated CSF protein levels in 30% of patients with neurological symptoms during the acute phase of COVID-19 (0–35 days after positive identification for SARS-Cov-2 PCR).^[26]^ Further, the CSF blood albumin ratio, which is indicative of BBB function, was elevated in 11 of 25 cases.^[26]^ Garcia et al reported Qlim(alb) < QAlb in 1 of 4 patients with acute encephalopathy who underwent CSF examination between 1 and 23 days (median 6 days) after a diagnosis of COVID-19.^[27]^ These studies demonstrate BBB function may be impaired during the acute phase of COVID-19.^[26,27]^ On the other hand, a multi-center study comprising 150 patients reported persistently increased CSF QAlb values for more than 30 days after the onset of neurological complications associated with COVID-19 in 55.6% of cases.^[28]^ Although the extent of inflammatory injury to the BBB and mechanisms of repair have yet to be fully elucidated, damage to the BBB during the acute phase of COVID-19 may remain for many months.^[28]^ Thus, there may be cases of encephalopathy after the fourth week of COVID-19 onset, similar to our cases. BBB failure is also considered a cause of septic encephalopathy (SE).^[29]^ SE typically presents with impaired consciousness, focal signs or myoclonus, and slow activity of the background rhythm or epileptic discharge on EEG^[29]^ as observed in the present case series. SE can be triggered by cytokine storm or systemic inflammation and is thought to be caused by increased permeability of the BBB due to NO or damaged tight junctions.^[29,30]^ Endothelial cell damage due to COVID-19 might represent a distinct pathogenic process from those underlying SE ^[29,30]^; however, residual BBB damage caused by COVID-19 might contribute to the onset of DE. The trigger of DE remained unknown in our series.

Long COVID-19, post-acute COVID-19, or chronic COVID-19 syndrome is characterized by fatigue and shortness of breath weeks or months after the onset of COVID-19.^[12–14]^ Long COVID-19 also causes neurological symptoms such as cognitive dysfunction, insomnia, olfactory and taste disturbances, which can last for several months.^[12–14]^ The DE we describe here was similar to long COVID-19 in that neurological symptoms appeared more than 4 weeks after the onset of COVID-19 without active pneumonitis.^[12–14]^ However, the neurological symptoms of DE were acute encephalopathy-like acute disturbances of consciousness or focal neurological deficits that improved during hospitalization, with no long-term symptoms other than tremors that lasted several weeks or months. This distinction in clinical courses suggested that DE was distinct from long COVID-19. In terms of patient demographics, while long COVID-19 affects both young and old people without regard to disease severity,^[22]^ all patients with DE were elderly and had moderate or severe COVID-19. Several factors appeared to be involved in the pathogenesis of long COVID-19, including prolonged pulmonary, neurological, cardiac, gastrointestinal, or immune dysfunction.^[12]^ Some studies have found a link between long COVID-19 and BBB failure;^[31,32]^ the pathogenesis of DE suggests BBB dysfunction but is not conclusive, and it is unclear whether this is due to the same pathogenesis as long COVID-19.

This report does have few limitations. This report is based on an examination of a small number of cases from a single institution. As a result, there may be similar cases that do not have all of the following features: recovery from moderate or severe COVID-19, acute encephalopathy after the fourth week of COVID-19 onset without exacerbation of pneumonitis, and accompanied by a tremor. Furthermore, data for non-DE cases are unavailable, and making comparisons is impossible. Prolonged BBB failure has been reported in patients with COVID-19 related neurological symptoms;^[28]^ however, it is unknown whether BBB failure exists in patients without neurological symptoms, and the cause of DE is also unknown. More research is needed to determine the pathogenesis and trigger of DE after COVID-19.

## 5. Conclusion

In patients recovering from moderate or severe COVID-19, DE occurred 4 weeks or later after the onset of COVID-19. DE symptoms included impaired consciousness, aphasia, apraxia, or hemiparesis, all of which were accompanied by a postural tremor. At the onset of DE, there was no recurrence of COVID-19 pneumonitis. Impaired BBB function due to COVID-19 appears to play an important role in the pathogenesis of DE after COVID-19.

## Acknowledgments

The authors would like to thank Enago (www.enago.jp) for the English language review.

## Authors contributions

**Conceptualization:** Takayoshi Akimoto, Makoto Hara, Tomotaka Mizoguchi.

**Data curation:** Takayoshi Akimoto, Kenta Tasaki, Yusuke Kurosawa, Tadaharu Nakamoto, Satoshi Hirose, Tomotaka Mizoguchi, Yuki Yokota.

**Funding acquisition:** Makoto Hara.

**Investigation:** Kenta Tasaki, Yusuke Kurosawa, Tadaharu Nakamoto, Satoshi Hirose, Tomotaka Mizoguchi, Yuki Yokota, Satoko Ninomiya.

**Methodology:** Takayoshi Akimoto.

**Supervision:** Makoto Hara, Hideto Nakajima.

**Visualization:** Takayoshi Akimoto.

**Writing – original draft:** Takayoshi Akimoto.

**Writing – review & editing:** Makoto Hara, Kenta Tasaki, Yusuke Kurosawa, Tadaharu Nakamoto, Satoshi Hirose, Tomotaka Mizoguchi, Yuki Yokota, Satoko Ninomiya, Hideto Nakajima.

## Supplementary Material


