# Supplementary material for: Delayed encephalopathy after COVID-19: A case series of six patients
Source: Medicine (Baltimore). 2022 Oct 21;101(42):e31029. doi: 10.1097/MD.0000000000031029 (PMC9592135; doi:10.1097/MD.0000000000031029)
Supplement: Supplementary file 1 [file medi-101-e31029-s001.pdf]

## Case presentation of each patient

Case 1.

A 65-year-old man with a history of hypertension and type 2 diabetes mellitus presented to our hospital six days after developing a sore throat and fever. Nasopharyngeal SARS-CoV-2 PCR testing was positive. Chest CT demonstrated scattered ground glass opacities in both lungs led to a diagnosis of moderate COVID-19. Treatment with dexamethasone, favipiravir, and nafamostat was commenced. He was discharged home on day 25. He reported the development of mental fatigue two days after discharge and erratic behavior including urinating on a heater four days after discharge. Accordingly, he was admitted to our hospital 37 days after the initial symptoms of COVID-19. Observations at the time of admission comprised a temperature of 37.3°C, $SpO_2$ of 97% (on room air), and a Glasgow Coma Scale (GCS) score of 12 points (E4V3M5). He demonstrated inattention and could not name objects. Generalized tremor and asterixis-like negative myoclonus were observed. Nasopharyngeal SARS-Cov-2 PCR testing was positive. Cerebrospinal fluid (CSF) examination demonstrated a CSF cell count of 18/$mm^3$ (all mononuclear cells) with a protein level of 115 mg/dL, IgG index of 0.45, IL-6 (Interleukin-6) level of 10.9 pg/mL, and oligoclonal bands. PCR testing of CSF was negative for herpes simplex virus (HSV), varicella zoster virus (VZV), and SARS-CoV-2. Qlim(alb) was calculated

as 8.3 and QAlb was calculated as 22.3. Serum D-dimer levels were within normal limits. In-house screening of neuronal antibodies in serum and CSF was negative. Chest CT demonstrated ground glass opacities in both lungs, which were improved compared to previous CT images. There were no abnormal findings on brain MRI. He was treated with acyclovir, steroid pulse therapy, and intravenous immunoglobulin therapy. Consciousness levels improved gradually and were almost normal on day 14. Electroencephalogram (EEG) performed on day 10 of second hospitalization demonstrated diffuse slowing. EEG on day 17 was normal background rhythm. He was discharged home on day 23 of the second hospitalization without any cognitive impairment; however, postural tremor persisted for approximately seven months.

Case 2.

A 68-year-old woman with a history of lung adenocarcinoma attended with a one-day history of cough and fever. Nasopharyngeal PCR testing for SARS-Cov-2 was positive, and she admitted to our hospital. On admission, there was no hypoxemia and plain chest radiography was normal. Treatment with dexamethasone and favipiravir was commenced. However, nine days after admission, she developed hypoxemia with a $SpO_2$ of 92% (on room air) and chest CT demonstrated ground glass opacities in both lungs. Accordingly,

she was diagnosed with moderate COVID-19 and treated with nafamostat, remdesivir, tocilizumab, and immunoglobulin. She was discharged on day 44 requiring home oxygen therapy. Twenty-three days after discharge (67 days after initial symptoms of COVID-19), she was brought to our hospital by her family due to inappropriate responses to questions and apraxia characterized by an inability to flush her toilet. Observations at the time of readmission demonstrated a body temperature of 37.2□, $SpO_2$ of 100% (on 2L oxygen), and a GCS score of 13 points (E4V3M6). She was able to state her name but unable to correctly state the date, place, or name familiar objects. Nasopharyngeal SARS-Cov-2 PCR testing was positive. Serum D-dimer levels were within the normal range. Laboratory blood testing demonstrated no abnormalities other than a serum anti-thyroglobulin antibody level of 264 U/mL. CSF examination demonstrated a cell count of 1/$mm^3$, protein level of 63 mg/dL, IgG index of 0.44, Qlim(alb) of 8.5, QAlb of 10.8, IL-6 level of 7.9 pg/mL, and absence of oligoclonal bands. PCR testing of CSF was negative for HSV, VZV, and SARS-Cov-2. Neuronal autoantibodies were not detected. Chest CT demonstrated no evidence of active pneumonitis. Brain MRI showed no abnormalities. She was treated with acyclovir, 500 mg/day of thiamine, and steroid pulse therapy. Four days after admission, she was able to name objects and no longer responded inappropriately to questions and steroid therapy was discontinued. On day nine of second

hospitalization, she had mild postural tremor affecting both upper limbs. On day 15, EEG demonstrated diffuse slowing with sporadic high-ampritude frontal delta activity. She was discharged on day 19. Twenty days after the second discharge, EEG demonstrated that background rhythm was normal, theta waves were reduced, and delta waves were disappeared. Her tremor persisted for five months after discharge. At 16 months after discharge from the encephalopathy, there was no evidence of lung cancer progression and no symptoms indicative of cancerous meningitis had appeared. In addition, brain MRI 15 months after discharge showed no findings suggesting metastasis.

Case 3.

A 72-year-old man with a history of rectal cancer attended with a one-day history of fever. Nasopharyngeal PCR testing for SARS-CoV-2 was positive. Chest CT demonstrated ground glass opacities in both lungs. He was diagnosed with moderate COVID-19 and treated with dexamethasone, nafamostat, and remdesivir. He was discharged on day 36 without sequelae. Fifteen days after discharge (51st day after initial COVID-19 symptoms), he was unable to speak and could only nod his head, suggesting motor aphasia. His speech recovered soon; however, he developed right facial weakness and hemiplegia and was brought to our hospital. At the time of admission, his

observations were as follows: temperature, 36.6°C; blood pressure, 113/77 mmHg; heart rate, 95/minute; $SpO_2$, 98% (on room air); and GCS, 14 (E4V4M6). Clinical examination demonstrated mild dysarthria and mild right hemiplegia. Serum D-dimer levels were elevated at 8.7 μg/ml. Head CT showed no abnormal findings. Chest CT demonstrated ground glass opacities in both lungs that were improved compared to the previous CT. He was diagnosed with ischemic stroke and antithrombotic therapy was started; however, brain MRI and MRA three days after admission showed no abnormalities. Hemiplegia improved after two days and dysarthria improved after five days. CSF examination on day 11 of second hospitalization demonstrated a cell count of 2/$mm^3$, protein level of 97 mg/dl, IgG index of 0.44, Qlim(alb) of 8.8, QAlb of 20.1, IL-6 level of 3.2 pg/ml, and negative SARS-Cov-2 PCR testing. Neuronal autoantibodies were negative. EEG on day nine of second hospitalization demonstrated diffuse slowing with frontal intermittent rhythmic delta activity (FIRDA). Postural tremor was observed in both upper limbs on day 11. He was discharged on day 37 without receiving immunotherapy. Postural tremor resolved two months after discharge. At 16 months after discharge from the encephalopathy, the rectal cancer had not progressed and no symptoms indicative of cancerous meningitis had appeared.

Case 4.

An 83-year-old man with hypertension, type 2 diabetes mellitus, and a history of ascending colon cancer attended with a one-day history of cough and fever. Nasopharyngeal SARS-Cov-2 PCR testing was positive. He developed hypoxemia with a $SpO_2$ of 93% (on room air) and ground glass opacities in both lungs. Accordingly, he was diagnosed with moderate COVID-19 and treated with dexamethasone, favipiravir, nafamostat, tocilizumab, and immunoglobulin. He was discharged 30 days after the onset of COVID-19. Sixteen days after discharge (47th day after initial symptoms of COVID-19), he developed sudden-onset erratic behaviors including stuffing clothes into a toilet bowl and plate biting and was brought to our hospital by his family. On admission, body temperature was 36.8°C, $SpO_2$ was 99% (on room air), and GCS score was 13 (E4V3M6). He was unable to state his own name or the names of familiar objects. No motor paralysis was observed. Nasopharyngeal SARS-Cov-2 PCR testing was negative. Serum levels of CRP (1.51 mg/dL) and D-dimers (1.8 μg/ml) were mildly elevated. CSF examination revealed a cell count of $1/mm^3$, protein level of 61 mg/dl, IL-6 level of 2.9 pg/ml, IgG index of 0.41, Qlim(alb) of 9.5, and QAlb of 11.9. PCR testing of CSF was negative for HSV, VZV, and SARS-Cov-2. CSF cytology was class I. Neuronal autoantibodies were not detected. Chest CT demonstrated improved findings of COVID-19 pneumonitis. Brain MRI showed no abnormalities. He was treated with acyclovir and 500 mg/day of

thiamine. He had diarrhea on admission and *Clostridium difficile* toxin was detected in stool. Accordingly, immunotherapy was not administered. He was able to state his name after one day of hospitalization, and state the correct date after four days of hospitalization. EEG after four days of hospitalization showed a diffuse slowing with FIRDA. On day 11 of second hospitalization, postural tremor was observed in both arms. He was discharged on day 19 of second admission. Two weeks after discharge, tremor had fully resolved. EEG at 52 days after discharge demonstrated normal background rhythm. Four months after discharge, the patient showed no neurological deterioration, and follow-up was terminated.

Case 5.

A 73-year-old woman with no previous medical history attended with a one-day history of fever and dyspnea. Nasopharyngeal SARS-Cov-2 PCR testing was positive and $SpO_2$ was 92% (on room air). Accordingly, she was admitted and treated with favipiravir and dexamethasone. However, hypoxemia worsened and extensive ground glass opacities were observed in both lungs on CT and remdesivir and tocilizumab therapy was initiated. Despite these treatments, hypoxemia did not improve and nasal high flow was commenced on day five. She developed respiratory distress syndrome and was diagnosed

with severe COVID-19. Endotoxin absorption therapy was provided on days 8 and 9 of admission and steroid pulse therapy from days 19 to 21 of admission. Hypoxemia gradually improved and she was transferred to another hospital for rehabilitation after receiving prednisolone on day 44. At the rehabilitation hospital, she exhibited disorientated on day 62 of initial COVID-19 symptoms. No abnormalities were observed on brain MRI. As her consciousness level did not improve, she was transferred to our hospital on day 65 of initial COVID-19 onset. Observations at the time of readmission were as follows: body temperature, 36.9°C; blood pressure, 133/92 mmHg; heart rate, 103/min; $SpO_2$, 93% (on room air), and GCS, 13 (E4V3M6). Her verbal comprehension was good; however, she was unable to speak in long sentences indicating motor aphasia. There was no motor paralysis. Postural tremor was observed in both upper limbs. Nasopharyngeal SARS-Cov-2 PCR testing was negative. Serum D-dimer levels were within normal limits. CSF examination revealed a cell count of $1/mm^3$, protein level of 51 mg/dl, IgG index of 0.42, Qlim(alb) of 8.9, QAlb of 9.9, and absence of oligoclonal bands. PCR testing of CSF was negative for HSV, VZV, and SARS-Cov-2 PCR. Neuronal autoantibodies were not detected. Chest CT demonstrated inflammatory fibrosis due to COVID-19 in both lungs with no new lesions. Acyclovir and steroid pulse therapy were started. On the second day after admission, her consciousness level improved and

her speech and writing became normal. EEG on day three demonstrated diffuse slowing with FIRDA. MRI performed on day seven demonstrated no abnormalities. After three days of steroid pulse therapy, prednisolone dosage was reduced. Mini Mental State Examination score was 28 on day 28. She was discharged home on day 29 of the second hospitalization. Postural tremor remained one months after discharge. EEG of 146 days after the second hospitalization demonstrated diffuse alpha background rhythm.

Case 6.

A 76-year-old right-handed man undergoing chemotherapy for small cell lung cancer presented to our hospital with a one-day history of fever and diarrhea. He underwent contrast-enhanced MRI one month prior to admission that showed no cancerous invasion of the central nervous system. Chest CT demonstrated multiple foci of ground glass opacities the left lung. Nasal COVID-19 PCR testing was positive and $SpO_2$ was 94% (on room air). Accordingly, he was diagnosed with moderate COVID-19 and treated with dexamethasone and favipiravir. He was discharged home without sequelae on day 25. Eighteen days after discharge (43 days after COVID-19 onset), he presented to our hospital with dysarthria and muscle weakness in both lower limbs. Observations at the time of admission were as follows: body temperature, 37.5°C; blood pressure, 142/79

mmHg; $SpO_2$, 96% (on room air); and GCS, 14 (E4V4M6). Severe stuttering and bilateral lower limb weakness were observed on clinical examination. Chest CT demonstrated pre-existing small cell lung cancer and resolution of ground glass changes. Brain MRI demonstrated an acute lacunar infarct in the right basal ganglia. Serum D-dimer levels were elevated at 1.9 μg/ml. CSF examination demonstrated a cell count of 2 $mm^3$, protein level of 61 mg/dL, IgG index of 0.39, Qlim(alb) of 9.1, QAlb of 12.7, IL-6 level of 4.5 pg/ml, and absence of oligoclonal bands. PCR testing of CSF was negative for SARS-Cov-2. Screening for neuronal antibodies was negative. CSF cytology was class II. EEG demonstrated diffuse slowing with FIRDA. He was diagnosed with cerebral infarction and treated with ozagrel sodium and clopidogrel. Dysarthria and stuttering gradually improved; however, tremor affecting both arms developed on day seven of second hospitalization. He was transferred to a rehabilitation hospital on day 20 of second hospitalization due to residual gait disturbance. His cancer status is unknown because he has not visited our hospital since his transfer.
